# MicroRNA-155-5p Diminishes in Vitro Ovarian Cancer Cell Viability by Targeting HIF1α Expression

**DOI:** 10.34172/apb.2020.076

**Published:** 2020-08-09

**Authors:** Ysrafil Ysrafil, Indwiani Astuti, Sumadi Lukman Anwar, Ronny Martien, Firasti Agung Nugrahening Sumadi, Tirta Wardhana, Sofia Mubarika Haryana

**Affiliations:** ^1^Departement of Pharmacology and Therapy, Faculty of Medicine, Public Health and Nursing, Universitas Gadjah Mada, Farmako Yogyakarta 55281, Indonesia.; ^2^Department of Surgery, Faculty of Medicine, Public Health and Nursing, Universitas Gadjah Mada, Farmako Yogyakarta 55281, Indonesia.; ^3^Departement of Pharmaceutics, Faculty of Pharmacy, Universitas Gadjah Mada, Yogyakarta, Sekip Utara Yogyakarta 55281, Indonesia.; ^4^Faculty of Pharmacy Universitas Muhamadiah Malang, Jl. Bendungan Sutami No. 188-A 65145, Malang, Indonesia.; ^5^Faculty of Medicine, Jenderal Soedirman University, Dr. Gumbreg, Mersi, Banyumas, Jawa Tengah 53112, Indonesia.; ^6^Medicine and Health Sciences of Doctoral Program, Faculty of Medicine, Public Health and Nursing, Universitas Gadjah Mada, Farmako Yogyakarta 55281, Indonesia.

**Keywords:** Mimics miR-155-5p, SKOV3, Ovarian Cancer, HIF1α

## Abstract

***Purpose:*** Ovarian cancer is the most lethal of gynecological malignancies. Recently, the development of microRNA (miRNA) -based therapeutics that could impact broad cellular programs, leading to inhibition of cancer cell viability, is gaining attention in the therapeutic landscape. The therapy is based on the presence of aberrant expressions of miRNA in cancer cells. Decreasing of tumor suppressor miRNA expression causes upregulation of oncoprotein, which worsens the prognosis of the ovarian cancer.

***Methods:*** miR-155-5p mimics were carried by chitosan nanoparticles using new nanotechnology methods. Cellular uptake of miRNA was assessed by fluorescence microscope while MTT and qPCR assay were used to determine miRNA profile and the effect of CS-NP/miRNA on SKOV3 cells.

***Results:*** Results of profiling validated using quantitative realtime-polymerase chain reaction (PCR) found one of the most altered tumor suppressor miRNAs, miR-155-5p was downregulated 892.15-fold. According to bioinformatic analysis we identified the miRNA could recognize and regulate HIF1α expression. Transfection of mimics for miR-155-5p showed significantly increased miR-155-5p endogen SKOV3 expression level compared to the control group. We found differences after transfection mimics for miR-155-5p 31.5 and 63 nanoMolar. Increasing of miR-155-5p endogen lead to diminished SKOV3 viability (by 30%; <0.05 at concentration 80 nanoMolar). These mimics may cause an increase in upregulated miR-155-5p endogen that can reduce HIF1α expression. Here we found 2-fold and 2.8-fold reduction of HIF1α expression level after transfection compared to the control group.

***Conclusion:*** According to these findings, the mimics miR-155-5p can inhibit ovarian cancer cell proliferation by regulating HIF1α expression.

## Introduction

Ovarian cancer is one of the most common of all gynecologic malignancies and represents one of the leading causes of cancer death for women. The disease has become a major health problem because of the increasing incidence rate every year.^1,2^ In 2012 GLOBOCAN estimated that 239 000 women were diagnosed with ovarian cancer with 152 000 women deaths worldwide annually, and some 600 000 women were living within five years of a diagnosis. The incidence steadily has increased, and in 2015 there were 251 000 new cases with about 161 000 women who died from the disease.^1,3^ Surgical resection of the tumor followed by platinum and texane based chemotherapy are the primary therapeutic interventions. Although the majority of patients respond to initial treatment, the 5-year survival rate remains only 30%. The condition is typically associated with more aggressive tumors, massive peritoneal dissemination and resistance.^4,5^

Recently researchers found that microRNAs (miRNAs), an emerging class of small non-coding RNAs are implicated in a wide variety of cellular processes including ovarian cancer. The worsening prognosis of this disease is also found to be related to miRNA expression patterns. MiRNAs are small non-coding RNA molecules (18-24 nt) that play roles in the regulation of target-gene expression post-transcriptionally by binding to complementary target mRNAs which results in mRNA translational inhibition or degradation.^6,7^ They have been widely implicated in pathogenesis of several human diseases, including cancers.^8^ Their aberrant expression in tumors have important pathogenetic consequences. MiRNAs that are overexpressed in tumors contribute to oncogenesis by downregulating tumor suppressors, while the tumor suppressor miRNAs that contribute to oncogenesis by downregulating oncogenic protein expression are downregulated. Recent studies found that one miRNA can regulate hundreds of target genes and play a role in global cellular physiology.^9,10^

For example, miRNA-21 is an oncomirRNA that can repress expression of tumor suppressor protein PDCD4 in colorectal cancer by recognizing and interacting with 3’UTR of messenger RNA.^11^ Another miRNA that becomes upregulated is miR 210 which modulates the expression of proteins involved with SHDH, a tumor suppressor that has a role in HIF1α expression and causes lung cancer cell survival.^12^ Binding of miR-10b in HOXD10 mRNA also initiates gene translation repression and leads to metastasis of breast and ovarian cancer.^13,14^

Contrarily, another important set of miRNAs that is downregulated in cancer is the tumor suppressor miRNAs that play roles in the repression of oncogenic protein translation.^9^ Decrease of these protein levels leads to cancer progression. For example, deletions of chromosomal loci in chronic lymphocytic leukemia lead to downregulation of tumor suppressor miRNAs, miR-15 and miR-16. They are important in chronic lymphocytic leukemia by targeting Bcl-2 mRNA and decreasing of their expression results in the development of an autonomous lymphoproliferative disorder.^15,16^ Another tumor suppressor miRNA that has received substantial attention is miR-34 which is involved in tumor cell cycle by regulation of CDK4 and CDK6 expressions, and also metastasis proteins such as MET, Notch, MYC and AXL expression.^17^

The importance of the role of miRNA in cancer is becoming more understood, and it is possible to be used as a therapeutic approach through the use of anti-miR which will modulate the oncogenic inhibition of miRNA and mimic miRNA to help endogenous miRNA suppress tumors by suppressing the oncogenic expression of proteins.^18,19^ Both of these therapeutic approaches have been shown to be effective *in vitro* and *in vivo* for several types of cancers such as prostate, breast, and colorectal cancer.^18-20^ Due to the nature of miRNA that is easily degraded in blood circulation and the difficulty in achieving its target when given systemically in the form of naked miRNA, it needs an appropriate vector.^7^ The strategy developed in the delivery of the miRNA to the site of action is by formulating it in nano-sized carriers. Nanoparticles are non-viral vectors which are often used in conveying genetic material because they are relatively safe to use and are able to protect the miRNA from nucleation degradation and deliver it into the intracellular compartment of the target cell.^21,22^ Chitosan is a cationic polymer that is often formulated as an oligonucleotide nanocarrier because it forms nano-sized and stable particles and forms complexes with oligonucleotides to carry them through cell membranes or tissues.^21,23,24^ Therefore, chitosan is considered capable of being used as a nanocarrier for the delivery of miRNA into cancer cells.

The purpose of this study was to characterize and evaluate the effect of chitosan nanoparticles miRNA-155-5p in alleviating ovarian cancer cell lines of SKOV3.

## Materials and Methods

### Materials 

The human epithelial ovarian cancer cell lines SKOV3 and Vero (normal cell line) were obtained from Stem Cell and Cancer Institute Kalbe (Jakarta, Indonesia). Ovarian cancer cell lines were grown in DMEM High Glucose (Massachusetts, USA) supplemented with 10% fetal bovine qualified serum (Massachusetts, USA), 1% Penicillin-Streptomycin (Massachusetts, USA), and 0.5% amphotericin (Massachusetts, USA). Exiqon miRCURY LNA^TM^ Universal RT microRNA PCR kit, miRCURY RNA Cell and Plant Kit, Universal cDNA synthesis kit II 8-64 rxns were purchased from Exiqon (Woburn, USA). Chitosan medium molecular weight were purchased from Sigma Aldrich (St. Louis, MO).

### Determination of miR-155-5p levels of normal and ovarian cancer cell lines by qRT-PCR

To determine the miRNA expression profiles of ovarian cancer cell line (SKOV3) and Vero cell line (normal), we used quantitative reverse transcription-polymerase chain reaction (qRT-PCR) in two panels of sample cells. The Exiqon miRCURY LNA^TM^ Universal RT microRNA PCR kit was used for all experiments. The procedure was conducted according to the manufacturer’s protocol. The miRNA expression was measured with Biorad CFX 96. Results of measurement were analysis by Biorad CFX Manager^TM^ and GENEX software.

### Chitosan nanoparticle-miRNA preparation

The nanoparticle formulation begins by dissolving the chitosan medium molecular weight powder into 1% acetic acid and stirring for 4 hours (with a magnetic stirrer). After adjusting pH with 1 M NaOH to pH 5.5, then the solution was regenerated with acetate buffer pH 5 so that 0.2% chitosan solution was obtained. Nanoparticles were made by ionic gelation method by mixing 0.2% chitosan with sodium tripolyphosphate (5:1) and incubating for 5 minutes at room temperature. 150 μL of the solution was conjugated with 150 μL of mimic miR-155-5p and then incubated for 20 minutes at room temperature.

### Efficiency entrapment

Efficiency entrapment of hsa-miR-155-5p microRNA encapsulation by chitosan nanoparticles was done by measuring the free concentration of hsa-miR-155-5p/antimiR-324-5p in the formula. Prepared nanoparticles were centrifuged for 15 minutes at a speed of 13,000 g. The supernatant obtained was measured by its absorbance using NANO-Quant (SPARK TECAN). Its absorption efficiency (%) was obtained from the percentage of encapsulated microRNAs (total miRNA-free miRNA) compared to the total RNA.

### In vitro cellular uptake

SKOV3 lines were seeded in 24 well plates at a density of 4 ×10^4^/well with borosilicate coated covered glass, followed by incubation. After 24-hours incubation, cells were transfected with chitosan nano-particle/FAM-conjugated miRNA-155-5p complexes with a concentration of miRNA 2 μM. Cellular uptake of miRNA was assessed by fluorescence microscopy (Carl Zeiss Axio^®^) 4 hours post incubation with miRNA labeled FAM. Prior to observation, cells were washed with DPBS to disperse the nanoparticles and then the cells were stained by DAPI to visualize nuclei.

### Cell viability assay

Cytotoxic tests were conducted by MTT Assay method using SKOV3 line cells. SKOV3 line cells (6000/well) were grown on a 96-well plate and incubated for 24 hours at 37^°^C and 5% CO_2_. The media was removed from the well plate, and the prepared nanoparticles (mixed with serum DMEM Free media) were put into the well (100 μL/well) then incubated for 24 hours at 37^°^C and 5% CO_2_. The test solution was removed and the well-plate was filled with 100 μL MTT 0.5 mg/mL. After incubation for 4 hours, 100 μL stop solution (SDS 10%, and 0.01 mol/L HCl) were added to dissolve formazan crystals and then were incubated again for 18 hours at room temperature in the absence of light. The absorbance of each well was measured at a 595 nm wavelength using a Micro Plate Reader (Bio-Rad Model 680 XR).

### Gene target prediction 

Gene target prediction was assessed to predict the complementary binding of microRNA-mRNA using starmiRDB and GeneCard.

Total RNA was isolated by miRCURY RNA Cell and Plant Kit, according to the manual protocol. RNA concentration was measured by a nanodrop at wavelengths of 260 and 280 nm. 10 ng of the total RNA in reverse transcription becomes cDNA according to the protocol kit used, namely Universal cDNA synthesis kit II 8-64 rxns. Quantification of miRNA-155-5p (5’-UUAAUGCUAAUC GUGAUAGGGGU-3’) was done using the ExiLent SYBR Green master mix kit, 2.5 mL (Cat No. 203402, Exiqon). miRNA-16-5p (5’-UAGCAGCACGUAAAUAUUGG CG-3’) was used as housekeeping gene.

Meanwhile, quantification of HIF1α mRNA expression used the following primers: (forward: 5’-AATGCTC CCCTCACCCAACG-3’; reverse 5’-GCAGGGTCAG CACTACTTCG-3) using SensiFASTTM SYBR^®^ kit (Cat. No. BIO-98020, Bioline) and β-actin (forward: 5’GGGAATTCAAAACTGGAACGGTGAAGG3’; reverse 5’GGAA GCTTATCAAAGTCCTCGGCCA CA-3 ‘) was employed as housekeeping gene. All gene transcriptions were quantified using the Biorad CFX 96 C.1000 quantitative PCR machine. Relative gene expressions were analyzed using the 2^-ΔΔCT^ method by Bio-Rad CFX Manager 3.0 and GenEx software version 6.1 and the results were expressed as the fold change.

### Statistical analysis

All measurements were done in triplicate and expressed as mean ± standard deviation (SD). Statistical analyses of miR-155-5p and HIF1α expression were performed using one-way ANOVA and to determine the presence of any significant differences between groups, followed by Bonferroni tests.All the data were analyzed using SPSS 16 software (SPSS Inc., Chicago, USA) and graphics were performed by GraphPad Prism 7.Statistical significance was set at *P* < 0.05.

## Results and Discussion

### Profile expression of miRNAs in ovarian cancer cell

Recently, the use of miRNA-based therapies as an approach in the treatment of diseases such as cancer and infections has been gaining attention. Aberrant expression of miRNA in ovarian cancer causes worsening prognosis of the cancer. This therapy aims to suppress the expression of oncogenic proteins by inserting mimic oligonucleotides.^10^

In this study, we determined there were many miRNA levels which were expressed showing differences between ovarian cancer cell line SKOV3 compared to normal cell line. We analyzed about 60 miRNAs from both of the SKOV3 and Vero cell lines that dysregulated (upregulated and downregulated). Of the 60 miRNAs, we found that miRNA-155-5p was the most common miRNA that appeared to be dysregulated (downregulated) about 892.15 times compared to the normal cell line (*P* < 0.05).

MiRNA 155 is a multifunctional microRNA located on chromosome 21q21.3 and involved in various biological and pathological processes in the body when it is disregulated.^25^ In ovarian cancer, this miRNA belongs to a group of tumor suppressor miRNAs that are able to regulate the regulation of the expression of some oncogenic proteins such as claudin-1 (CLDN1) which is important for the invasion and adhesion of ovarian cancer-initiating cells.^26^ In addition, this miRNA can directly recognize and inhibit XIAP expression and promote apoptosis in ovarian cancer cells SKOV3, A2780, and primary cultured ovarian cancer cells. Furthermore, the administration of miR-155-5p expression on ovarian cancer cells can increase the sensitivity of cisplatin to ovarian cancer cells.^25^ Downregulation of its expression causes the ability to carry out its duties in suppressing protein oncogenesis to be reduced and causes worsening prognosis of the cancer.

### Evaluation of miRNA-155-5p transfection by chitosan nanoparticle

**Figure 1:**
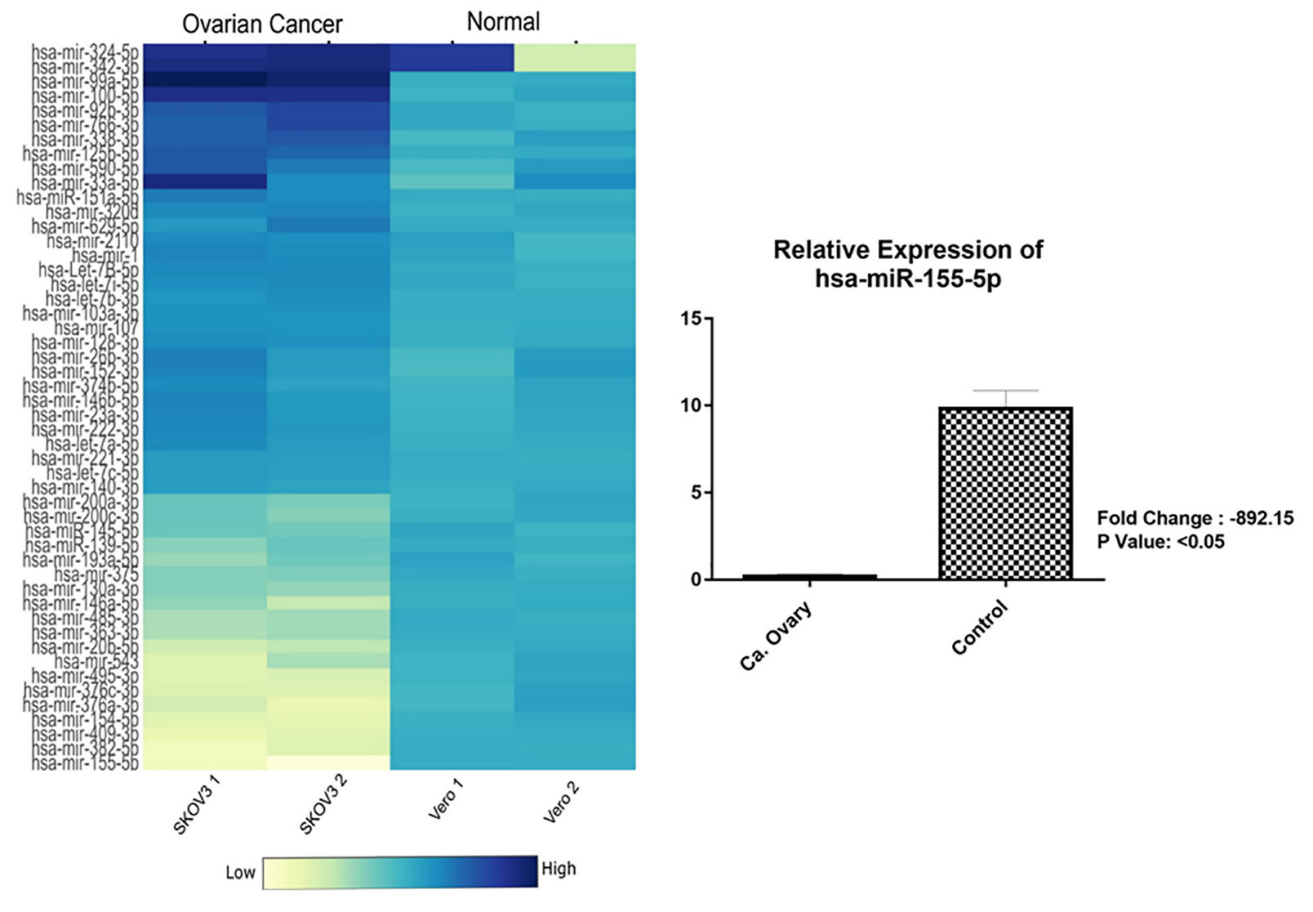
Differentially expressed miRNAs. **a**. heat-map representation of expression patterns of the differentially expressed miRNAs in ovarian cancer cell line versus Vero cell line. Rows of the heat map represent miRNAs; columns show profiled cancer and the control (normal) group comprises Vero cell line. Relative expression is represented as a colorgram (Blue: high expression). **b**. The expression of miRNAs as determined by qPCR analysis. Shown are comparisons of the average relative expression levels of hsa-miR-155-5p in ovarian cancer cell line and Vero cell line (*P* < 0.05).

To further show transfection efficiency was investigated by FAM detection of fluorescence microscopy (Carl Zeiss Axio^®^) and miR-155-5p expression level by qPCR. As seen from the arrows in Figure 1, green fluorescence of FAM labelled miR-155-5p were detected at 4 h post-incubation in cytoplasmic of SKOV3 cell, while the naked miR-155-5p had no fluorescence after 4 h post-incubation.

**Figure 2:**
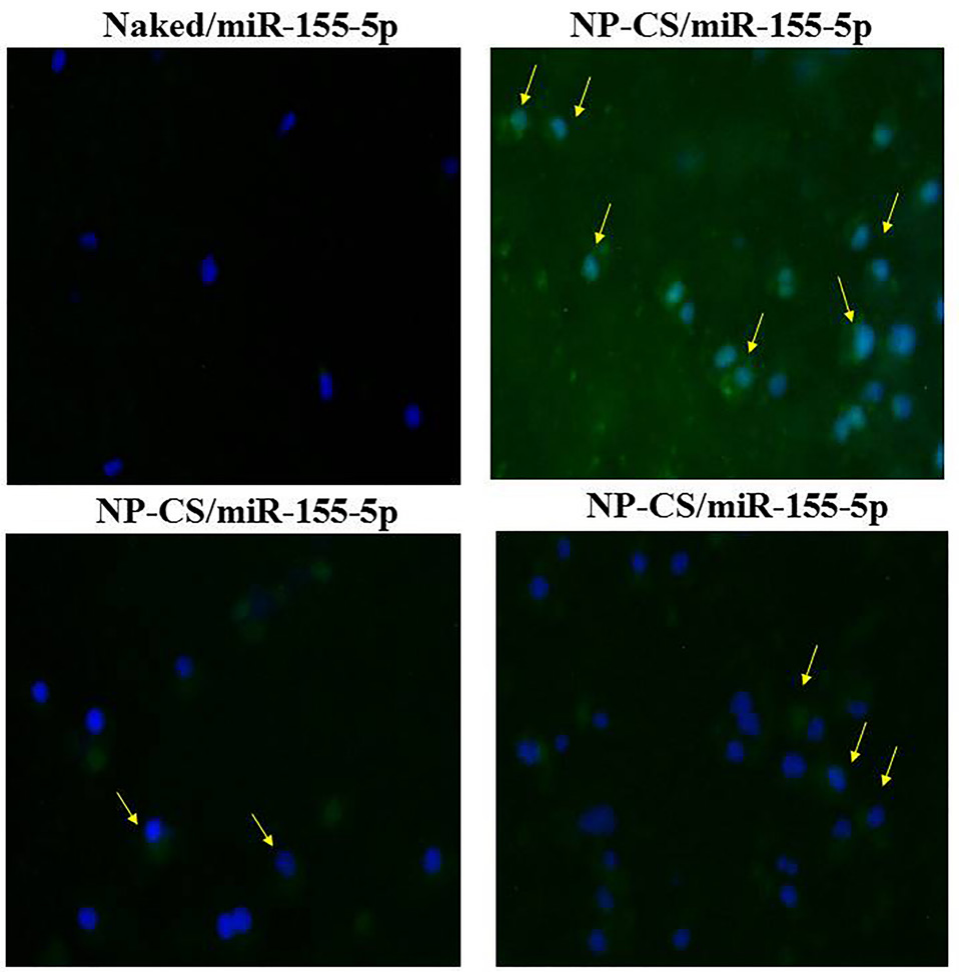
Cellular uptake localization of chitosan/FAM-miR-155-5p mimic nanoplexes in SKOV3 cell lines after 4 h incubation by fluorescence microscopy.

According to previous cellular uptake study by chitosan/FAM mimic miR-155-5p and antimiR-324-5p found that release miRNA might occur within the first 4 h for most of the tested chitosans. Furthermore, to access the uptake cellular of mimic miR-155-5p, we also measured endogenous miR-155-5p level by qPCR (Figure 2).

### Efficiency entrapment

**Figure 3:**
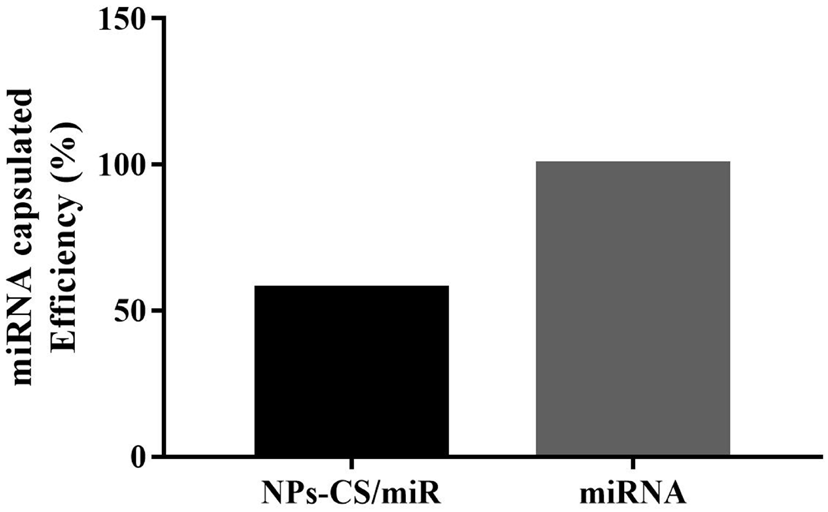
The efficiency of the mimic miR-155-5p encapsuled in chitosan nanoparticles measured by NANO-Quant (SPARK TECAN).

The encapsulation efficiency of microRNA by chitosan can be seen in Figure 3. The efficiency of encapsulation in nanoparticles is very important in the therapeutic effect of microRNA delivered using a nanocarrier system. This efficiency value refers to the amount of microRNA that is absorbed in the chitosan matrix through two mechanisms namely the ionic interaction of the (-NH_3_^+^) group with a negative charge of miRNA and tripolyphosphate of crosslinker. In the figure it appears that 57.43% of miRNA-155-5p is absorbed in the nanochitosan. This entrapment efficiency is influenced by chitosan concentration. The low concentration of chitosan will result in a low chitosan viscosity which also causes the miRNA to penetrate more quickly into the polymer matrix.^27^

**Figure 4:**
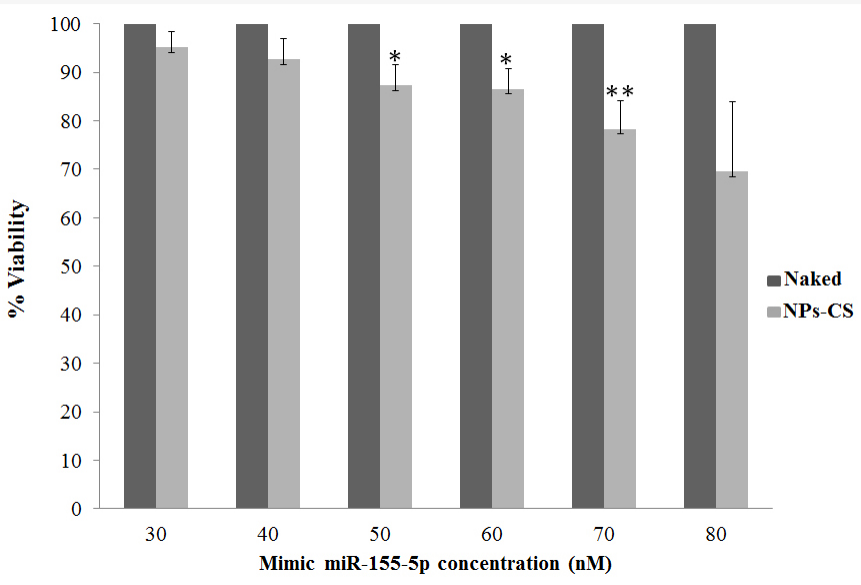
Inhibition Proliferationof chitosan nanoparticle of mimic miR-155-5p to ovarian cancer cell line SKOV3 (n=3). The persentage are mean ± SD. *P < 0.05 and **P < 0.005 versus naked mimic miR-155-p. Abbreviations: Ch-NPs, chitosan nanoparticles.

The absorption of microRNAs in this chitosan matrix enables miRNA-155-5p protection from the degradation of the nuclear enzyme and is easily delivered to the target action to carry out its function in silencing the expression of target genes and initiating inhibition of ovarian cancer cell proliferation. This is evidenced by the presence of microRNA luminescence labeled FAM (green) in the cytoplasmic region of SKOV3 cells (Figure 2). This luminescence was not found in groups of cells that were transfected without chitosan. Besides this finding, it is also proven by the results of cytotoxic test between miR-155-5p expression without and encapsulated chitosan in Figure 4. In the figure, it appears that the administration of mimic miRNA without conjugated miRNA does not have an inhibitory effect on ovarian cancer cell proliferation compared to the mimic miRNA that was conjugated with nanoparticles chitosan (Figure 4).

### Effect of miRNA-155-5p on viability of SKOV3 cell 

Cell viability study of nanoplexes were conducted using the mitochondrial dehydrogenase performance measurement, also known as the 3-(4,5-dimethyl-2-thiazolyl)-2,5-diphenyl-2H-tetrazolium bromide (methyl thiazolyl tetra-zolium; MTT) assay (Figure 4). Our findings show significant inhibition on cell viability after treated by mimic miR-155-5p compared to control (*P* < 0.05).

MTT assay is a colorimetric method in cytotoxicity test that is used because it is simple. This method is based on mitochondrial dehydrogenase activity in cytochrome b and c from living SKOV3 cells that can cut the tetrazole ring of MTT and are reduced to form purple formazan crystals. This substance is easily dissolved in dimethyl sulfoxide or other organic impurities.^28^ The assay was done with several variations of the concentration of mimic oligonucleotide and anti-miRNA namely 30 nM, 40 nM, 50 nM 60 nM 70 nM and 80 nM. This increased percentage of inhibition of cell death was not seen in the treatment group given microRNA without chitosan.

This finding is likely due to the administered microRNA being degraded before reaching the target action so that it cannot carry out its function in suppressing the expression of the target gene. Inhibition of cell growth in the test group treated with miRNA chitosan nanoparticles is an indication that the microRNA has successfully entered the SKOV3 cell and performs its function in suppressing the expression of target genes (e.g XIAP and CLDN1) and initiating cancer cell death.

### In silico miRNA target prediction

**Figure 5:**
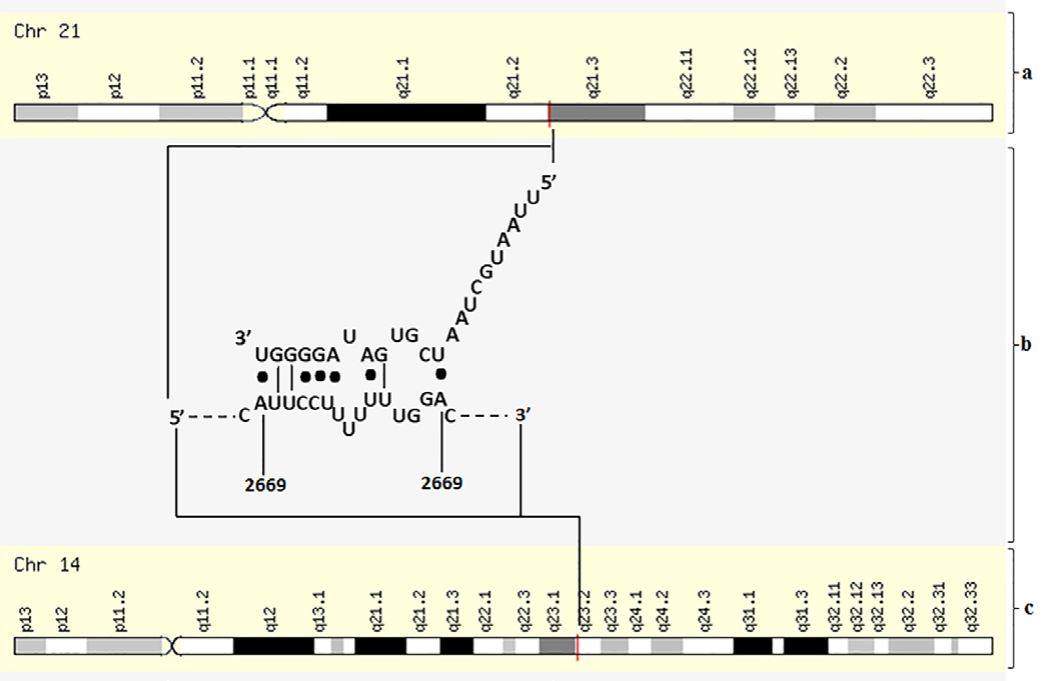
In silico prediction of hsa-miRNA-155-5p-HIF1α-3′UTR recognition and interaction is highly favorable and almost certain to occur in the cytoplasm /nucleoplasm of SKOV3 cells by  using computational tool StarMirDB combine with GeneCard. (a,c) hsa-miR-155-5 and HIF1α location in chromosome, (b) hsa-miRNA-155-5p-HIF1α-3′UTR interaction prediction.

Furthermore, our bioinformatics analysis found that miRNA-155-5p that acts as a tumor suppressor miRNA in ovarian cancer can recognize HIF1α at 2655-2669 base with logistic probability of 0.854 and ΔG_total_ -13.39 kcal/mol (Figure 5).

### Effect of mimic miRNA-155-5p on endogenous miRNA-155-5p and HIF1α expression level on SKOV3

**Figure 6:**
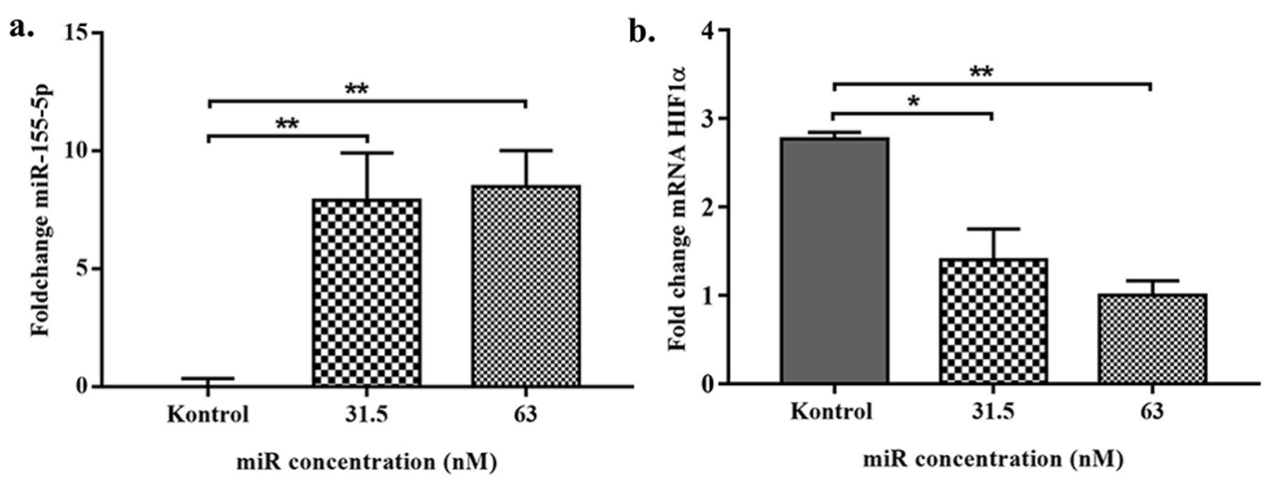
Fold change of expression endogenous miRNA-155-5p (A) and mRNA HIF1α SKOV3 cell line post treathment NPs-CS mimics miRNA-155-5p 31,5 nM and 63 nM. Expression of miRNA and mRNA were measured by qPCR and analyzed using the 2-^ΔΔCT^ method Fold change are mean ± SD *P < 0,05; **P < 0,01 versus control group (SKOV3 cell line untreated).

To assess whether the nanoparticles were able to deliver mimic miRNA-155-5p in a functional location on SKOV3, we measured the miR-155-5p level after 24-hour incubation by qPCR and compared the results with the control cells. As seen in Figure 6A, there were significant differences of miRNA-155-5p level between the control and chitosan nanoparticle miR-155-5p in both of the 31.5 nM and 63 nm groups in the SKOV3 cell line. Furthermore, we confirmed that transfected mimic miRNAs were functional miRNA by measuring the target of miRNA, such as HIF1α mRNA expression as the one of the miR-155-5p direct targets as shown in Figure 6B.

Furthermore, we found that expression levels of miR-155-5p were significantly upregulated after 24-hour transfection of chitosan nanoparticle mimic miRNA-155-5p (*P* < 0.05) (Figure 5A). Increasing of level endogenous miR-155-5p SKOV3 lead to poor viability of SKOV3 cell line (Figure 4). Assess of miRNA level does not accurately report the level of functional miRNA transfected because it does not distinguish between miRNAs in functional or non-functional pools. To assess that the transfected miRNAs are functional, we evaluated the biological effects of miRNA-155-5p on silencing the target gene. As seen from *in silico* analysis results miR-155-5p is a tumor suppressor miRNA that can recognize and interact with oncogenic post transcription gene HIF1α. Furthermore, to confirm that entered mimic miRNAs have biology effect, we measured the HIF1α mRNA expression.^29^ Previously a negative correlation was reported between miRNA-155-5p expression level and mRNA HIF1α in ovarian cancer serum.

According to Zhu et al HIF1α was expressed in SKOV3 cell line. In this study, we found that increasing of miR-155-5p levels can reduce HIF1α expression level.^30^ The decrease of HIF1α levels are statistically significant to control group vs 31.5 nM and 63 nM treatment group. This is consistent with the findings of Bruning et al that showed application of miR-155 can reduce the expression of HIF-1α mRNA, protein and transcriptional activity in hypoxia.^31^

Hypoxia-inducible factor 1-alpha is a master regulator of oxygen homeostasis by inducing glycolysis, erythropoiesis, and angiogenesis. The protein is commonly upregulated in human cancers as survival mechanism and in their metastases. The proteins demonstrated the ability to transcribe some oncogenic proteins such as VEGF, ABCB1 and GLUT-1 that play critical roles in the survival and metastasis of ovarian cancer cells.^32^

## Conclusion

In summary, we concluded that chitosan nanoparticle could be used as a delivery system for anti and mimic miRNA. Furthermore, *in vitro* study revealed that miRNA transfection of mimic-155-5p that conjugated with chitosan nanoparticle can induce inhibition of viability of ovarian cancer cell line SKOV3 by decreasing HIF1α.

## Ethical Issues

The article does not contain any study with human and animal subject performed by any of the authors.

## Conflict of Interest

Authors declare that there is no conflict of interest.

## Acknowledgments

This study was supported financial by PTUPT (1818/UNI/DITLIT/DIT-LIT/LT/2018) and PPUPT (1987/UNI/DITLIT/DIT-LIT/LT/2018) grants from the Ministry of Research, Technology, and Higher Education-Republic of Indonesia.
